# The Progress in Diagnosis and Treatment of Exosomes and MicroRNAs on Epileptic Comorbidity Depression

**DOI:** 10.3389/fpsyt.2020.00405

**Published:** 2020-05-12

**Authors:** Nian Wei, Haiqing Zhang, Jing Wang, Shen Wang, Wenbo Lv, Limei Luo, Zucai Xu

**Affiliations:** ^1^Zunyi Medical University, Zunyi, China; ^2^Department of Neurology, Affiliated Hospital of Zunyi Medical University, Zunyi, China; ^3^Prevention and Health Care, Affiliated Hospital of Zunyi Medical University, Zunyi, China; ^4^Key Laboratory of Brain Science, Zunyi Medical University, Zunyi, China

**Keywords:** exosomes, microRNAs, epilepsy, depression, diagnosis, treatment

## Abstract

The occurrence of epilepsy can increase the incidence of depression, and the risk of epilepsy in the patients with depression is also high, both of which have an adverse effect on the life and the psychology of the patient, which is not conducive to the prognosis of the patients with epilepsy. With lucubrating the function of exosomes and microRNAs, some scholars found that the exosomes and its microRNAs have development prospect in the diagnosis and treatment of the disease. MicroRNAs are involved in the regulation of seizures and depression, as biomarkers, that can significantly improve the management of epileptic patients and play a preventive role in the occurrence of epilepsy and epilepsy depressive disorder. Moreover, due to its regulation to genes, appropriate application of microRNAs may have therapeutic effect on epilepsy and depression with the characteristics of long distance transmission and stability of exosomes, to a certain extent. This provides a great convenience for the diagnosis and treatment of epileptic comorbidity depression.

## Introduction

Epilepsy is a clinical syndrome of highly synchronous abnormal discharge of brain neurons caused by many causes (1). About 65 million people worldwide suffer from epilepsy which is the common nervous system disease (2). The occurrence of epileptic diseases will not only cause health problems, but also have a serious impact on patients’ mood, occupation, life and overall quality of life. Epilepsy patients have a large psychological pressure and are often associated with various mental disorders, especially depression. Depression is the most common mental complications in epileptic patients, with population—based studies suggesting that one in three epilepsy patients may suffer from depression (3, 4). The disability rate and mortality of epilepsy accompanied by depression are higher than that of non-depression group, and the effect of antiepileptic drugs and surgery is worse than that of non-depression group (5). Therefore, the diagnosis and treatment of epileptic co-depression is urgent. Nowadays, there are more and more researches linking the pathophysiology of seizures and depression to exosomes and microRNAs (6–8).

Exosomes are a class of membrane lipid vesicles with a diameter of between 40 and 100 nm (9), containing messenger RNAs, microRNAs, proteins, and liposomes, which can involve in cell-to-cell communication and targeting cells (10). Exosomes can be widely extracted in the blood, urine, cerebrospinal fluid, and other bodily fluids (11, 12). And the number and composition of exosomal miRNAs are different between epileptic or depressive patients and healthy individuals (13). MicroRNAs are a type of highly conservative with a length of about 22 nucleotides noncoding single-stranded RNAs encoded by an endogenous gene (14), which can participate in the regulation of gene expression by that a single miRNA target hundreds of mRNAs. And microRNAs can be excreted by exosomes which offer a stable environment for secreted microRNAs (15). The exosome containing microRNAs flows out of the cerebrospinal fluid and can cross the blood-brain barrier, so that a biological marker representing a brain disease can be extracted in the peripheral blood (16), such as epilepsy (17) and depression. In addition, the pathological mechanisms of epilepsy and depression are complex and may involve blood-brain barrier injury, central nervous inflammatory response, demyelinating lesions, nerve cell injury, etc (18, 19). Exosomes, as a new type of cell communication vector, can participate in the regulation of central nervous system neuroinflammation by specific binding with target cells of nervous system, reduce neuronal loss and so on (20). For example, researchers treated pilocarpine-induced epilepsy models with stem cell-derived exosomes and found that this exosomes alleviated inflammation and improved status epilepticus-induced learning and memory impairment in mice by targeting hippocampal astrocytes (11).

Therefore, we herein review the development of exosomes and microRNAs in the field of epilepsy and depression, as well as the current clinical diagnosis and treatment in this neighborhood. And the objective of this paper is to explain the prospect of exosomes and microRNAs in the diagnosis and treatment of epilepsy comorbid depression, and to seek new ideas for the clinical diagnosis and treatment of epilepsy comorbid depression.

## The Current Clinical Diagnosis and Treatment of Epilepsy Comorbid Depression

Epilepsy is a central nervous system disease, chronic brain disease characterized by transient central nervous system dysfunction caused by abnormal discharge of brain neurons. There has been a problem with the definition of epilepsy, which has recently been elaborated by ILAE as a disease of the brain defined by any of the following conditions: 1) At least two unprovoked (or reflex) seizures occurring >24 h apart; 2) one unprovoked (or reflex) seizure and a probability of further seizures similar to the general recurrence risk (at least 60%) after two unprovoked seizures, occurring over the next 10 years; 3) diagnosis of an epilepsy syndrome (2). And the new definition is designed to increase clinical operability to ensure diagnostic accuracy (2). According to statistics, the prevalence of epilepsy in developed countries accounts for 0.5%–1% of the total population, and is higher in developing countries (21). Domestic epidemiological data show that the total prevalence of epilepsy in China is 7%, and it increases at the rate of 400,000 people per year (22). Depression has become a common and serious comorbidity in epilepsy patients, and data show that the prevalence of depression in epilepsy patients reaches 22.9% (23), which is higher than the prevalence of 6.1%–9.5% in the general population (24). For children with epilepsy, the prevalence of this comorbidity is even more frightening, reaching 21% to 60%, 3–6 times higher than for the general population (25, 26). Epilepsy comorbid depression further exacerbates seizures and has a far greater adverse effect on their quality of life than seizure frequency and severity (27). At present, there is no systematic, standardized diagnostic criteria for epilepsy comorbidity depression. Its clinical diagnosis mainly depends on the Neurological Disorder Depression Inventory for Epilepsy (NDDI-E) as a rapid and effective clinical detection, but the characteristics of the self-assessment of the scale may affect the evaluation results owe to the patient’s sense of disease shame (28). While in comorbidity treatment, some antiepileptic drugs (such as phenobarbital) that reduce the levels of serotonin and norepinephrine in the synapse can lead to depressive episodes (29), and SSRIs (Selective serotonin reuptake inhibitors)antidepressants can increase the risk of seizures (30). In addition, In addition, due to the complexity and heterogeneity of the etiology of epilepsy, some patients with seizures are difficult to control, and about 30% to 50% of patients with refractory epilepsy are accompanied by depression (31). Therefore, the diagnosis and treatment of epilepsy comorbid depression needs further exploration.

## MicroRNAs, Biomarkers for Epilepsy and Depression, Facilitate the Prognosis of Epileptic Comorbidity Depression

### MicroRNAs as Potential Diagnostic Biomarkers for Epilepsy

The occurrence of epilepsy involves neurotransmitter signals, ion channels, synaptic structures, neuronal death, glial hyperplasia, and inflammation (32). MicroRNAs is a small class of non-coding RNAs, that can regulate gene expression (33). And the regulation by microRNA networks can involve in the development of human brain (34), so the breaking of dynamic balance may lead to neuropathology, such as epilepsy. Raoof et al. did a follow-up study, they performed genome-wide PCR-based and RNA sequencing in plasma from a larger cohort of samples (> 250) from two countries, drawing a conclusion that miR-27a-3p, miR-328-3p and miR-654-3p could be regarded as potential biomarkers for epilepsy (35). Moreover, these microRNAs levels in exosomes consisted in plasma are higher, that provides the feasibility of these microRNAs as a potential diagnostic marker for epilepsy patients (36). On the other hand, for genetic generalized epilepsies, it may be more connection with microRNAs. Martins‐Ferreira et al. found that miR-146a, miR-155, and miR-132 may partake in genetic generalized epilepsies(GGE) epileptogenesis and they reported that the three circulating microRNAs have potential value to be GGE biomarker (37). In particular, the striking up-regulation of miR-132 in epilepsy has been described in several experimental articles (38–40). Xiang et al. overexpressed miR-132 in the hippocampal neuronal culture model of status epilepticus with transfection technique, they manifested that the overexpression of miR-132 significantly increases the frequency of epileptic discharge in epileptic neurons, that is similar to that miR-132 participates in the postepileptic enhancement of high voltage activated Ca2+ currents (41). These discoveries suggest that miR-132 may play an epileptic role in the development of epilepsy. However, what’s interesting is that miR-132 can be regarded as a negative regulator of transforming growth factor beta 1 (TGF-β1) and cytokine interleukin‐1 beta(IL-Iβ) induced epileptogenic factors (39). And previous studies have found that the actived TGF-β1 encourage epileptogenesis (42, 43). Likewise, the proinflammatory pathway mediated by cytokine interleukin-1 (IL-Iβ) participates in the occurrence of epilepsy (44). Alyu et al. proposed that IL-Iβ is associated with epileptic seizures by that it can enhance the ability of astrocytes to release glutamate and reduces glutamate reuptake, thereby increasing the utilization of glutamate in neuronal synapses and promoting the overexcitability of neurons (45). In conclusion, miR-132 plays an important role in the development of epilepsy, but its regulation mechanism needs further study.

### MicroRNAs as Potential Diagnostic Biomarkers for Patients With Depression

Nowadays, the incidence of depression is getting higher and higher, and the disorder of epilepsy with mental disorder is also the majority of the depression. Early detection and early diagnosis are beneficial to the prognosis of depression. More and more studies indicate that many microRNAs express to be abnormal in patients with depression. For example, a standpoint that microRNA-124 can act as a biomarker for depression has been put forward in experiments involving rodents, human postmortem brain, and blood cells of depressed patients (46, 47). Roy and Dunbar et al. constructed a CORT-induced depression rats model, they found that the expression of miR-124 was upregulated in PFC, and then the consistent result was validated in post-mortem brain samples of depression patients, moreover, the similar dysregulation of miRNA-124 was detected in peripheral blood serums of depression patients who were psychotropic drug-free for at least one month (47). The maladjustment of hypothalamus–pituitary–adrenal (HPA) axis can increase depression prevalence in epilepsy patients (48), and the sustained overactivity of the HPA axis is a manifestation of the brain’s inability to response depression state (49). And the expression of Nr3c1 and Gria4 associated with stress responsive pathway in depression can promote the activity of HPA axis, however micro-124 has inhibitory effect on these two genes (46). Moreover, there is evidence that the overexpression of miR-124 in hippocampus enhanced the adaptability to depression-like behavior and the decrease of hippocampal miR-124 could boost the sensitivity to depression-like behavior after normal benign mild chronic stress regimen (50). In addition, some scholars compared the expression profile of various microRNA in patients with depression with that in normal subjects, from the results of their comparison, the expression of the microRNA let-7b and let-7c was significantly down-regulated in patients with depression, and let-7c and let-7b participate in regulation for the expression of 27 genes which involved in the PI3k-Akt-mTOR signaling pathway (51, 52). However, one of the 27 genes is the insulin-like growth factor (IGF1) which is a significant over-representation in the regulation of let-7c and let-7b, and which can promote the signal conduction of the brain-derived neurotrophic factor, and can also be combined with the brain-derived neurotrophic factor to induce the anti-depression effect (52). Besides, Gheysarzadeh et al. found that the serum levels of three miRNAs (miR-16,miR-135a, and miR-1202) in patients with depression who were medication−free for at least 2 months before sampling were significantly lower than those in normal subjects, these miRNAs can also be potential biomarkers for depression (53). And what calls for special attention is that microRNAs may be affected by antidepressants, such as mentioned above miR-16 whose important targets cover serotonin transporters, and in mouse model, chronic utilization with the SSRI fluoxetine can increase levels of miR-16 in serotonergic raphe nuclei (54). Similarly, management of acute or chronic SSRI antidepressants in a mouse model of depression can augent miR135a levels in the raphe nuclei (55). Unfortunately, there was no significant difference between miR-16 and miR135a levels in human blood before and after treatment with SSRI antidepressants (55). Nevertheless, Issler and other researchers measured levels of miR135a in the blood of depression patients after three months of cognitive behavioral therapy (CBT), exposing a prominent up-regulation in miR135a levels after CBT (55). Of course, there is a lot of research on the effect of antidepressant therapy on miRNA expression in depression patients (56, 57), for instance, miR124 levels in peripheral blood of depressed human patients decreased significantly after 8 weeks of treatment with various antidepressants (58). In conclusion, it is promising that microRNAs are regarded as biomarkers for the diagnosis of depression or the evaluation of antidepressant treatment.

## Exosomes Are Expected to Be a Nano-Therapeutic Agent for Depression of Epilepsy

### Exosomes Can Regulate Myelin Sheath Formation to Combat Epileptic Depression

Some people have done a postmortem pathological study on suicidal patients with severe depression, they found that oligodendrocyte density and the expression of oligodendrocyte function related genes are reduced for these patients, and they drew a conclusion for that patients with severe depression put up severe white matter demyelination (59–61). There are evidences that olig2 can promote the differentiation of oligodendrocyte progenitor cell (OPC) into myelin oligodendrocytes (62–64). And LINGO-1 is a negative regulator of myelin formation of oligodendrocytes in the central nervous system (65, 66). Whereas, a research manifested that Olig2 expression was decreased while LINGO-1 expression for the animal model with spontaneous recurrent epileptic seizures combined depression (SRS-D), and compared to normal group, Olig2 and LINGO-1 expression was no significant difference in the animal model with spontaneous recurrent epileptic seizures combined no depression (SRS-ND) (67). Meanwhile, the SRS-D group detected decreased myelin basic protein (MBP) expression and decreased myelination, likewise, the SRS-ND group had no significant difference with normal group (67). They consider that the demyelination disorder is associated with depression (68) and the demyelination in the epilepsy leads to the occurrence of the combined depression of the epilepsy (67). Fortunately, the exosomes have shown a positive effect in some other demyelinating diseases, exhibiting enhanced myelination and inhibition of demyelination. An animal experiment on the role of exosomes secreted by human mesenchymal stem cells (MSC) in the treatment of multiple sclerosis found that demyelination was reduced in exosome-treated experimental autoimmune encephalomyelitis (EAE) mouse (69, 70). So the exosomes secreted by MSCs have the effect of resistance to demyelination. Besides, myelination is a complex process which is regulated by micro RNAs, such as miR-219 and miR-338 (71, 72). Milbreta et al. applied a scaffolding system which enables sustained non-viral delivery of microRNAs to oligodendrocytes, and they found that the animals treated with miR-219/miR-338 preserved a higher number of Olig2, than the control group (73). They also authenticated caffold-mediated delivery of miR-219/miR-338 could enhance myelin formation after spinal cord injury (SCI) which can inhibit myelin formation by a phenomenon that MBP expression was more extensive in scaffolds that incorporated miR-219/miR-338 (73). Similarly, maybe transfection of miR-219/miR-338 into synthetic multivalent antibodies retargeted exosome which can control cellular immunity (74) to enhance axonal remyelination after nerve injuries in the central nervous system (CNS) to treat epileptic depression.

### Mesenchymal Stem Cell-Derived Exosomes and miR-132 May Treat Epileptic Comorbid Depression by Reducing Inflammation of Central Nervous System

The occurrence of epilepsy involves complex nervous system responses. The inflammation of the brain can promote the epilepsy, the activity of the seizure can promote the production of the inflammatory molecules, thus affecting the severity of the epilepsy and the frequency of the recurrence (75, 76). In the cerebrospinal fluid of the patients with epilepsy, the pro-inflammatory cytokines IL-Iβ have increased significantly, suggesting that the IL-Iβ level plays an important role in the occurrence and progression of epilepsy (77, 78). What’s interesting is that the IL-Iβ plasma levels in patients with temporal lobe epilepsy and depression are significantly higher than the levels in the people for temporal lobe epilepsy without depression, it has verified that there is positive correlation between IL-1 β level and depression (79) owing to that the rise of IL-1β in chronic temporal lobe epilepsy further upregulates Indoleamine 2,3-dioxygenase1 (IDO1) expression to increase the kynurenine/tryptophan ratio and ruduce the serotonin/tryptophan ratio in the hippocampus (80). This also reflects the role of IL-Iβ in epileptic depression. Besides, the evidence suggests that IL-1β knock-down in the hippocampus can significantly alleviate the memory deficits and anxiety and depression-like behavior of the mice induced by lipopolysaccharide(LPS) may due to eliminates the down-regulation of LPS-induced neuropeptide(VGF) and brain-derived neurotrophic factor(BDNF) (81). These evidences indicate that IL-1β is not only involved in the occurrence of epilepsy, but also promotes the occurrence of depression in patients with epilepsy. Nevertheless, an experiment on therapeutic effects of mesenchymal stem cell-derived exosomes (MSC-Exos) on retinal detachment found that the expression of IL-1β were significantly reduced after MSC-Exos treatment (82). The anti-inflammatory effects of MSC-Exos were also demonstrated in an animal experiment to improve the prognosis of sepsis syndrome (SS). First, they confirmed that SS causes a severe inflammatory response not only in circulation but also in the brain, leading to severe brain damage, they found that some inflammatory biomarkers (TLR-2, TLR-4, MYD88, IL-1β, TNF-α, NF-κB, and MMP-9) were significantly higher in cerebrospinal fluid from SS animals than control group, and the anomalies of these molecules in circulatory levels and in brain tissue as well as in CSF were observably inhibited by adipose-derived mesenchymal stem cell-derived exosomes (83). The anti-inflammatory effect of MSC-Exos, especially the inhibition of IL-1β, may have a certain treatment prospect for epileptic comorbidity depression. Moreover, exosomes has been considered to be a vector that promotes inter-cell communication and regulates the cell function by delivering proteins, RNA, and other molecular components, with its nature for biocompatibility, stability in the circulation, biological barrier permeability, low immunogenicity, and low toxicity (7, 84). And *in vitro* model experiments have shown that overexpression of miR-132 can reduce the expression of IL-Iβ (39, 85). In addition, it was reported that IL-1β could induce disruption of the blood-brain barrier (86–88). Blood–brain barrier dysfunction is related with epilepsy (89) and the experiment found that the indicator of blood–brain barrier dysfunction (90) (MMP-9 concentration) was significantly elevated after the seizure (91, 92). Similarly, the increased CSF to serum levels of peripheral markers including albumin and urate in depressed patients indicates a compromised blood-brain barrier (53). And the brain endothelium can express high levels of tight junction proteins and adherens junction molecules to ensure the integrity of the blood-brain barrier (93). Yet, exogenous miR-132 can suppress the expression of MMP-9 to protect the integrity of the blood-brain barrier by reducing degradation of tight junction proteins (94, 95). From the above, miR-132 can play a certain role in the treatment of epilepsy by anti-inflammatory and protecting the integrity of the blood-brain barrier. An experiment by Teng Ma et al. has used Gene Pulser II system to load miR-132 into exosomes, by that to form the miR-132-overexpressed exosomes nano-therapeutic (96). However, Mateos et al. have testified that miR-132-overexpressing therapy can exacerbate neuronal damage by an experiment that intracerebroventricular injection of an antagomir of miR-132 protected against hippocampal CA3 neuronal death 24 h after seizure (38). The pity is that the author did not consider the later time point after seizure when brain inflammation is more obvious (39). So, the optimal time window is a challenge for the miR-132-overexpressed exosomes nano-therapeutic.

Perhaps, the rational application of exosomes and miR-132 is effective to improve the prognosis of epileptic patients and reduce the incidence of depression.

## The Application Prospect of the Exosomes as a Nano-Therapeutic Carrier

Exosomes are formed by such a process that first endosome is took shape though invagination of cell membrane, then the endosome evolves to multivesicular bodies, last multivesicular bodies combine with plasmalemma reducing the release of intraluminal vesicle to extracellular. It carries their contents which contain specific mRNAs, regulatory microRNAs, lipids, cytokines and proteins (97–99) from the donor cell to the recipient cell for the purpose of altering the function of the target cell (100). Therefore, exosomes play a key role in long-range signal transduction between cells (101). And Exosomes have been reported to have a natural targeting ability based on donor cells owing to their inherent biological activity that they intrinsically express some lipids and cell adhesion molecules and ligands (102). So for their function, Luan et al. proposed that using technique to insert the gene encoding the targeting proteins into the donor cells to make the donor cells secreta a kind of exosomes which contain this proteins (102). For example, Ohno et al. applied exosomes in delivering let-7a miRNA in a targeted manner to breast cancer cells in mice (103). Similarly, combined with the above, using exocrine to deliver miR-219/miR-338 to CNS in a targeted manner may improve epileptic depression. In addition, a lot of efforts have also been made to develop exocrine bodies into carriers of drug transport. A variety of techniques have been reported for loading therapeutic agents which contain microRNA, protein, medicine, etc into the exosomes, and these techniques conclude sonication, extrusion, freeze and thaw cycles, electroporation, incubation with membrane permeabilizers, and click chemistry method for direct conjugation (102). Moreover, exosomes have prominent advantages as gene therapy delivery carrier for that they consist of cell membranes with multiple adhesion proteins on the surface (104). Besides, exosomes can cross major biological barriers such as the blood–brain barrier for their small size and flexibility (105). Compared to other carriers, exosomes are lower toxic because they are naturally secreted vesicles (106), and exosomes are more tolerated in the body for that they are ubiquitous in body fluids (107, 108). Of course, there are some challenges on the treatment of epileptic comorbidity depression in exosomes. The problem of the more accurate purification of exosomes and the mass production of exosomes in clinic still need our efforts (109), not to mention the further exploration of the relationship between exosomes and depression of epilepsy comorbidity.

## Summary and Perspectives

At present, epilepsy patients with depression disorder is common, especially those intractable epilepsy, they are mostly accompanied by depression. Fortunately, research on exosomes and microRNAs as a biomarker for epilepsy and depression is becoming more mature. Nevertheless, there is still a long way to apply microRNAs to the clinic. The detection of microRNAs in peripheral blood is more convenient than that in CSF in the clinical, but this ignores a problem whether these differences can really represent brain-derived genetic alterations. Maybe, it is a better alternative to measure the change of microRNAs in brain-derived exosomes in peripheral blood. And the techniques for quantifying microRNAs (such as qRT-PCR) need to be improved in terms of cost and speed. In addition, in consideration of a study on the molecular mechanism of the interaction of the depression of the epilepsy, such for IL-Iβ, it is promising to use exosomes as carrier in the treatment of epileptic comorbid depression. Moreover, exosomes not only play a potential role in the treatment of epileptic comorbid depression as a carrier, but also have great hope for the treatment of epileptic comorbid depression by exosomes themselves, especially MSC-Exos. However, equally, it remains a challenge that exosomes and microRNAs are applied to treat epilepsy comorbid depression in clinical. Most of the above studies have been conducted in animal models and *in vitro* experiments, whether these conclusions can be extended to the human body requires more exploration, but it is also an inspiration for seeking new ways to treat epilepsy comorbid depression. And the mechanism of epilepsy comorbidity depression needs to be further studied, and the pharmacodynamics and toxicology of exosomes *in vivo* need to be further explored.

## Author Contributions

NW, HZ and JW designed and written this article, NW, HZ, JW, SW, WL, LL, and ZX contributed to collection and analysis of literature. ZX helped with proofreading and revision. All authors read, revised, and approved the final manuscript.

## Funding

This research was supported by the grants of National natural science foundation of China (Grant No 81660227) and the Science and Technology Project in Guizhou Province (Grant No. QKHLHZ[2016]NO.7476).

## Conflict of Interest

The authors declare that the research was conducted in the absence of any commercial or financial relationships that could be construed as a potential conflict of interest.
